# Specialised functions of two common plasmid mediated toxin-antitoxin systems, *ccdAB* and *pemIK*, in *Enterobacteriaceae*

**DOI:** 10.1371/journal.pone.0230652

**Published:** 2020-06-30

**Authors:** Alma Y. Wu, Muhammad Kamruzzaman, Jonathan R. Iredell

**Affiliations:** 1 Centre for Infectious Diseases and Microbiology, The Westmead Institute for Medical Research, The University of Sydney, Westmead, New South Wales, Australia; 2 Westmead Hospital, Westmead, New South Wales, Australia; University of Graz, AUSTRIA

## Abstract

Toxin-antitoxin systems (TAS) are commonly found on bacterial plasmids and are generally involved in plasmid maintenance. In addition to plasmid maintenance, several plasmid-mediated TAS are also involved in bacterial stress response and virulence. Even though the same TAS are present in a variety of plasmid types and bacterial species, differences in their sequences, expression and functions are not well defined. Here, we aimed to identify commonly occurring plasmid TAS in *Escherichia coli* and *Klebsiella pneumoniae* and compare the sequence, expression and plasmid stability function of their variants. 27 putative type II TAS were identified from 1063 plasmids of *Klebsiella pneumoniae* in GenBank. Among these, *ccdAB* and *pemIK* were found to be most common, also occurring in plasmids of *E*. *coli*. Comparisons of *ccdAB* variants, taken from *E*. *coli* and *K*. *pneumoniae*, revealed sequence differences, while *pemIK* variants from IncF and IncL/M plasmids were almost identical. Similarly, the expression and plasmid stability functions of *ccdAB* variants varied according to the host strain and species, whereas the expression and functions of *pemIK* variants were consistent among host strains. The specialised functions of some TAS may determine the host specificity and epidemiology of major antibiotic resistance plasmids.

## Introduction

Toxin-antitoxin systems (TAS) were originally discovered on bacterial plasmids in the 1980s, [1, 2] but have since also been recognised on bacterial chromosomes [3]. TAS cassettes typically consist of two gene loci, governed by a common regulation mechanism [4], encoding a stable toxin that induces cell death or arrests growth, and a labile antitoxin that neutralises the toxin through binding to the toxin or other means. While the toxin is always a protein, the antitoxin can be protein or RNA based, and thus TAS can be categorised into six different types (Types I-VI), based on the nature and mechanism of action of the antitoxin [5, 6]. The Type II system, in which both toxin and antitoxin are proteins, is the typical model of a TAS and the best studied, and is probably the most common in bacteria [7].

It has long been known that plasmid-mediated TAS play a role in plasmid maintenance through postsegregational killing and/or inhibition of growth of plasmid free cells [2, 8, 9], while chromosomal TAS have been found to be involved in a range of other roles, such as in the bacterial stress response, antibiotic tolerance, persister cell formation, biofilm formation, bacterial virulence and intestinal colonisation [10–14]. However, several recent studies identified the involvement of plasmid-mediated TAS in a range of bacterial physiology beyond plasmid maintenance. We have recently showed that the plasmid-mediated *parDE* TA system is involved in antibiotic and heat tolerance in *Escherichia coli* [15]. Some other plasmid-mediated TA systems such as *ccdAB* contributes antibiotic persistence in *E*. *coli* [16], while *mpvAT* is involved in virulence by maintaining virulence plasmids in *Shigella* and *Salmonella* species [17, 18]. Within *Enterobacteriaceae*, TAS are common among conjugative plasmids including antibiotic resistance (AbR) plasmids, and often associated with certain plasmid incompatibility (Inc) types. For example, the type II TAS *pemIK* and *vagCD* are usually found on IncF and IncL/M plasmids, and IncF and IncHI2 plasmids respectively [19, 20].

The distribution of TAS in the plasmids found in *Escherichia coli* is well described [21–25], but there has been as yet no comprehensive study of the TAS in all known plasmids residing in *Klebsiella pneumoniae* species. Existing studies of a collection of clinically relevant *K*. *pneumoniae* genomes (encompassing both plasmids and chromosomes) has provided insights into the distribution of TAS in the chromosomes of this species and the association of some TAS with certain plasmid replicon and antibiotic resistance regions, suggesting possible strain specific specialisation of TAS, with some being localised to one species while others are found across species [26, 27]. Previous studies have also identified two distinct groups of *vagCD* TAS on plasmids located in *K*. *pneumoniae* [28], with each of the toxins and antitoxins shown to be functional. *vagCD* is a member of the large TA family *vapBC*, with the toxin VagD being a PilT N-terminal (PIN) domain containing endoribonuclease that inhibits translation [29, 30].

TAS with identical names infer similar roles, regardless of the initial source and sequence variations, but functional differences in TAS from different genetic contexts have previously been noted [31, 32]. For example, IncF plasmid-mediated *ccdAB* TAS (*ccd*_*F*_) from pathogenic *E*. *coli* O157:H7 is involved in post-segregational killing but its chromosomal counterpart (*ccd*_*O157*_) is unable to mediate PSK [32]. However, common TAS functions (*e*.*g*. plasmid maintenance or antibiotic tolerance) for plasmid-mediated TA variants were not investigated.

In this study, we analysed the distribution of type II TAS in 1063 fully sequenced plasmids found in *K*. *pneumoniae* retrieved from GenBank. Variation in DNA sequence, expression and function of two common TAS found in both *E*. *coli* and *K*. *pneumoniae* plasmids, *ccdAB* and *pemIK*, were examined.

*ccdAB* is a well-studied, primarily plasmid-associated type II TAS, although copies have also been found on bacterial chromosomes where it appears to be involved in the bacterial stress response [33, 34]. The toxin, CcdB, binds to and disrupts the action of DNA gyrase, causing double strand DNA breaks and the induction of the bacterial SOS response [35, 36]. *pemIK*, another well studied type II TAS that is related to the *mazEF* system, is an mRNA endoribonuclease that inhibits protein synthesis [37]. Here, we provide vital information about the similarity, specificity and functions of these two TAS, with broad implications for their role in the spread of antibiotic resistance.

## Materials and methods

### Identification of TAS on *K*. *pneumoniae* plasmids

The names and complete sequences of all plasmids from *K*. *pneumoniae* species available in GenBank at the time of search (August 2019) were retrieved (https://www.ncbi.nlm.nih.gov/genome/?term=klebsiella+pneumoniae), and the sequences of all plasmids >30 kb examined with TA Finder (http://202.120.12.133/TAfinder/TAfinder.php) [38] using the default parameters to identify potential type II TAS. Plasmids <30 kb in size were excluded as smaller plasmids generally do not carry TA systems. The plasmid incompatibility (Inc) type of each plasmid was defined using PlasmidFinder (https://cge.cbs.dtu.dk/services/PlasmidFinder/) [39].

### Alignment of TAS sequences

Representative examples of the two most common TAS, *ccdAB* and *pemIK*, were chosen, and the nucleotide and amino acid sequences of the toxin and antitoxin coding regions retrieved from GenBank (https://www.ncbi.nlm.nih.gov/genbank/) using coordinates obtained from TA Finder. To verify that these sequences were in fact representative of their specific variant, each of the sequences were used as queries for nucleotide Basic Local Alignment Search Tool (BLAST) searches (https://blast.ncbi.nlm.nih.gov/Blast.cgi) [40]. The resulting matches were compiled for each TAS, then aligned in MEGA7 (http://www.megasoftware.net/mega7/) [41] using the ClustalW algorithm, followed by construction of phylogenetic trees using the Maximum Likelihood method.

Variants were compared with one another by nucleotide alignment as described above. The amino acid sequences were then used to predict the secondary structures of the proteins using PsiPred (http://bioinf.cs.ucl.ac.uk/psipred/) [42].

TAS promoters were predicted using BPROM (http://www.softberry.com/berry.phtml?topic=bprom&group=programs&subgroup=gfindb) (Softberry) with default parameters, with the input being the 500 bp region upstream of the ATG start codon of the antitoxin gene. The putative promoter sequences were then aligned as described above.

### Plasmids, bacteria, primers and culture conditions

Tables 1, 2 and 3 list plasmids, bacterial strains and primers respectively. Bacteria were grown in Luria-Bertani (LB) broth (BD Biosciences, NJ, USA), with kanamycin (50 μg/mL) or chloramphenicol (20 μg/mL) (Sigma-Aldrich, MO, USA) added as indicated. Insertion of TAS into vectors was carried out using standard restriction digestion and ligation cloning protocols, and chemical transformation and electroporation into host strains was also performed using standard protocols. The plasmid construct details can be found in Table 1. Each solution used was rendered sterile either through autoclaving or filter sterilising at 0.22 μm.

**Table 1:** Plasmids used in this study.

Plasmid	Genotype or characteristics	Source
pANT3	Low copy, Kan^R^_,_ promoterless *gfpmut3*	[43]
pANT5	Low copy, Kan^R^, *gfpmut3* under *ptac* control	[43]
pACYC184	Low copy, Cm^R^, Tet^R^, cloning vector	New England BioLabs, Ipswich, USA
pBCSK+	High copy, Cm^R^, cloning vector	Catalog# 212215, Stratagene, CA, USA
pJIE134	Naturally occurring IncF plasmid from *E*. *coli*	unpublished
pJIE203	Naturally occurring IncF plasmid from *K*. *pneumoniae*	unpublished
pEl1573	Naturally occurring IncM plasmid	[44]
pJIAW07	Promoter region of *ccdAB* from pJIE134 inserted into the *Bam*HI/*Xba*I sites upstream of *gfpmut3* in pANT3 (“ccdAB-EC prom”)	This work
pJIAW15	Promoter region of *ccdAB* from pJIE203 inserted into the *Xba*I site upstream of *gfpmut3* in pANT3 (“ccdAB-KP prom”)	This work
pJIAW09	Promoter region of *pemIK* from pEl1573 inserted into the *Bam*HI/*Xba*I sites upstream of *gfpmut3* in pANT3 (“pemIK-LM prom”)	This work
pJIAW10	Promoter region of *pemIK* from pJIE134 inserted inserted into the *Bam*HI/*Xba*I sites upstream of *gfpmut3* in pANT3 (“pemIK-F prom”)	This work
pJIAW12	*ccdAB* with its own promoter and ribosome binding site (RBS) from pJIE134 inserted into the *Bam*HI site of pACYC184 (“ccdAB-EC low”)	This work
pJIAW16	*ccdAB* with its own promoter and RBS from pJIE203 inserted into the *Xba*I/*Hind*III site of pACYC184 (“ccdAB-KP low”)	This work
pJISP01	*pemIK* with its own promoter and RBS from pEl1573 inserted into the *Xba*I site of pACYC184 (“pemIK-LM low”)	This work
pJISP02	*pemIK* with its own promoter and RBS from pJIE134 inserted into the *Xba*I site of pACYC184 (“pemIK-F low”)	This work
pJIMK57	*ccdAB* with its own promoter and RBS from pJIE134 inserted into the XbaI site of pBCSK+ (“ccdAB-EC high”)	This work
pJIAW17	*ccdAB* with its own promoter and RBS from pJIE203 inserted into the BamHI site of pBCSK+ (“ccdAB-KP high”)	This work
pJIMK71	*pemIK* with its own promoter and RBS from pEI1573 inserted into the BamHI site of pBCSK+ (“pemIK-LM high”)	This work
pJIMK59	*pemIK* with its own promoter and RBS from pJIE134 inserted into the BamHI site of pBCSK+ (“pemIK-F high”)	This work

**Table 2:** Bacterial strains used in this study.

Strain	Genotype or characteristics	Source
***E*. *coli* strains**		
DH5α	Host strain used for cloning. F- ɸ80*lac*ZΔM15 Δ(*lac*ZYA-*arg*F)U169 *rec*A1 *end*A1 *hsd*R17(r_k_^-^mk^+^) *pho*A *sup*E44 *thi*-1 *gyr*A96 *rel*A1 *deo*R	Invitrogen (Carlsbad, USA)
BW25113	Laboratory strain of *E*. *coli* K-12	[45]
Ec WH62	Antibiotic sensitive, plasmid-free clinical *E*. *coli* isolate	[46]
Ec WH59	Antibiotic sensitive, plasmid-free clinical *E*. *coli* isolate	[46]
Ec WH67	Antibiotic sensitive, plasmid-free clinical *E*. *coli* isolate	[46]
***K*. *pneumoniae* strains**		
Kp ATCC13883	*K*. *pneumoniae* ATCC13883	American Type Culture Collection (ATCC)
Kp WH49	Antibiotic sensitive, plasmid-free clinical *K*. *pneumoniae* isolate	[46]
Kp WH81	Antibiotic sensitive, plasmid-free clinical *K*. *pneumoniae* isolate	[46]
Kp WH84	Antibiotic sensitive, plasmid-free clinical *K*. *pneumoniae* isolate	[46]

**Table 3:** Oligonucleotide primers used in this study.

Name	Sequence (5ʹ – 3ʹ)	Amplicon (bp)	Specificity	Reference or source (GenBank Acc.)
ccdFPRO-F1 ccdFPRO-R1	CGCGGATCCGCGGTAATTACGCTTTGTTT CCGTCTAGAAGCACACCTCTTTTTGACA	183	Promoter of *ccdAB* from *E*. *coli* IncF plasmids	EU418925.1
ccdKPPRO-F3 ccdKPPRO-R2	TTATCTAGACCGCTCAGCACCGGTAAA CGCTCTAGAACTGTTATGTCGCCATTAAT	202	Promoter of *ccdAB* from *K*. *pneumoniae* plasmids	Unpublished sequence
pemLMPRO-F pemLMPRO-R	CGCGGATCCGCTGGGTTTACTGTTTGGCT CCGTCTAGATGTTCACCTCCATAAAAG	114	Promoter of *pemIK* from IncL/M plasmids	JX101693.1
pemFPRO-F pemFPRO-R	CGCGGATCCCGCTGGGTTTACTGTTTGGT CCGTCTAGATCTTCACCTCCATAAAAGT	115	Promoter of *pemIK* from IncF plasmids	EU418925.1
Gfpjnx-2	GTTCTTCTATTTACTCAT	Various	For confirmation and orientation of inserts in pANT3	[43][43][43]
ccdF-F-BamHI ccdF-R-BamHI	ACAGGATCCACGAAACGGGAATGCGGTAA GCTGGATCCATGACTGCAGACTGGCTGTGT	761	Whole *ccdAB* system from *E*. *coli* IncF plasmid	EU418925.1
ccdKP-F3-HindIII ccdKP-R2-XbaI	ATAAAGCTTCCGCTCAGCACCGGTAAA GCGTCTAGATGCGGCAATGCTTCGTTTT	862	Whole *ccdAB* system from *K*. *pneumoniae* plasmid	Unpublished sequence
pemLM-F-XbaI pemLM-R-XbaI	GCTCTAGACGCGCTGGGTTTACTGTTTT GCTCTAGACAGGCATGTGACAACGCAGA	797	Whole *pemIK* system from IncL/M plasmids	JX101693.1
pem-F-XbaI pem-F-XbaI	GCTCTAGAAGAACTGTTCCTGGTGGGGTTG GATCTAGAAGAATGGTGGGACAACAGC	873	Whole *pemIK* system from IncF plamids	EU418925.1
pACYC184-F pACYC184-R	TTACGCGCAGACCAAAACGA GCGATATAGGCGCCAGCAAC	Various	For confirmation and orientation of inserts in pACYC184	New England BioLabs, Ipswich, USA
ccdF-XbaI ccdR-XbaI	GCTCTAGACGAAACGGGAATGCGGTAA AGTCTAGACATGACTGCAGACTGGCTGTGT	761	Whole *ccdAB* system from *E*. *coli* IncF plasmid	EU418925.1
pemF-BamHI pemR-BamHI	ACAGGATCCagaactgttcctggtggggttg GCTGGATccagaatggtgggacaacagc	873	Whole *pemIK* system from IncF plamids	JX101693.1
pemF1-BamHI pemR1-BamHI	ACAGGATCCGCGCTGGGTTTACTGTTT GCTGGATCCAGGCATGTGACAACGCAGA	797	Whole *pemIK* system from IncL/M plasmids	EU418925.1
ccdKpF-BamHI ccdKpR-BamHI	ACAGGATCCAACGGCCGTCCTGTAATTTAACG GCTGGATcCTGCGGCAATGCTTCGTTTT	770	Whole *ccdAB* system from *K*. *pneumoniae* plasmid	Unpublished sequence

Abbreviations: F, Forward; R, Reverse.

NB—Underlined bases indicate alterations in sequence to introduce *Bam*HI (G/GATCC), *Xba*I (T/CTAGA) or *Hind*III (A/AGCTT) restriction sites for cloning purpose

### Measurement of relative promoter strength

Relative strengths of putative TAS promoters were determined from TA promoter-*gfp* constructs using methods described previously [47]. Briefly, the predicted TAS promoters were cloned upstream of a promoterless *gfp* in the expression vector pANT3, and four strains each of *E*. *coli* and *K*. *pneumoniae* were transformed with these constructs. Overnight cultures were inoculated from single colonies into LB broth with kanamycin, and grown with shaking at 37°C. The cultures were then diluted 200 x in LB broth and grown under the same conditions for a further 3–4 h. Cells were harvested by centrifugation and the pellets resuspended in 0.85% sodium chloride (saline). The concentrations of each sample were then standardised to OD_580_ of 1.0 (~ 8.0 x 10^8^ cfu/mL), as determined using the DensiCHEK^TM^ Plus nephelometer (bioMérieux, France). Fluorescence was analysed using a Victor3 plate reader (Perkin Elmer, MA, USA), with an excitation wavelength of 485 nm and an emission wavelength of 535 nm. Experiments were performed in triplicate, and readings were averaged and corrected for background fluorescence by subtracting the pANT3 (no promoter) reading from each sample. The positive control pANT5, which constitutively expresses GFP from the *ptac* promoter, is a derivative of pANT3 [43]. Both plasmids carry the pRSF1010 origin of replication, which is a low copy broad-host range IncQ plasmid [48].

### Plasmid stability assays

To assess plasmid stability, the whole TAS (including the putative promoter and ribosome binding site) was cloned into a low and a high copy plasmid (pACYC184 and pBSCK+ respectively) and two strains each of *E*. *coli* and *K*. *pneumoniae* were transformed with these constructs. Plasmid stability was assessed as described previously [15]. Briefly, a single colony of *E*. *coli* or *K*. *pneumoniae* bacteria carrying the relevant plasmid was grown in LB broth at 37°C with shaking at 225 rpm for 72 or 96 hours, without antibiotic selection. Bacterial cultures were transferred into fresh LB medium at 1:1000 dilution at 16, 24, 40, 48, 64 and 72 hrs for high copy plasmids. Additional dilutions were performed at 88 and 96 hrs for low copy plasmids, as these are not lost as quickly as the high copy plasmids. Samples were taken before every transfer, diluted in saline and plated on to LB agar without antibiotic and incubated at 37°C for 18 h. From each plate, 120 colonies were replica plated onto LB agar plates with and without the indicator antibiotics to estimate plasmid retention.

## Results and discussion

### Distribution of type II TAS in the plasmids found in *K*. *pneumoniae* strains

Twenty-seven different putative type II TAS were identified (Table 4), with *ccdAB*, *pemIK* and *vagCD* most common among them (S1 Table). We compared the distribution of the TAS identified here in *K*. *pneumoniae* plasmids with previously reported TAS in *E*. *coli* plasmids [21]. *E*. *coli* and *K*. *pneumoniae* are closely related members of the *Enterobacteriaceae* family, sharing a large number of mobile antibiotic resistance genes, mainly via plasmids. However, only three TAS (*ccdAB*, *pemIK*, and *vagCD*) were common in both, with *ccdAB* and *pemIK* most commonly shared. Many, such as *MNT-HEPN*-like, *GNAT-RHH*-like, *Bro-Abr-*like and *Bro-ArsR-*like TAS appear to be most important in *Klebsiella* plasmids and have not previously been reported in *E*. *coli* plasmids [21, 23].

**Table 4:** Summary of TAS found on *K*. *pneumoniae* plasmids.

Plasmid type	No. of plasmid	Type II TA Systems*
IncF	568	None**, *vagCD*, *higBA*, *ccdAB*, *pemIK*, *GNAT-RHH* like, *MNT-HEPN* like, *COG5654-5642*, *relE-PhD* like, *relE-Xre* like, *pfam13420-TIGR01764*, *mazEF*, *mazF-RHH* like, *vapBC*, *parE-relB*, *relBE*, *MNT-RHH* like, *hicAB*, *fic-PhD* like, *relE-COG2442* like, *relE-COG5606* like, *COG12446-pfam13384*
IncR	76	*pfam13420-TIGR01764*, *vagCD*, *ccdAB*, *MNT-HEPN* like, *relE-Xre* like, *mazEF*, *mazF-AbrB* like, *relBE*, *pemIK*, *higBA*, *mazF-RHH* like, *GNAT-RHH* like, *COG5654-COG5642*
IncA/C	62	None, *pemIK*, *relE-Xre* like, *pfam12658-pfam00126*, *vagCD*, *GNAT-RHH* like
IncX	60	None, *hicAB*, *relE-Xre* like, *pfam13420-TIGR01764*, *vagCD*, *relBE*
IncH	58	*hipA-Xre* like, *relBE; COG5654-COG5642; GNAT-RHH* like, *relE-PhD* like, *relE*-*Xre* like, *MNT-HEPN* like, *vagCD*, *pemIK*, *pfam13420-TIGR01764*, *pfam12568-pfam00126*
IncL/M	51	None, *pemIK*, *pfam13420-TIGR01764*
IncN	33	None, *vagCD*, *MNT-HEPN* like
IncI	10	None, *hicAB*, *pfam13420-TIGR01764*
IncQ	7	*higBA*, *mazF-RHH* like, *pfam12568-pfam00126*, *relE-Xre* like, *GNAT-RHH* like, *pemIK*, *vagCD*, *COG12446-pfam133844*
IncU	2	None
IncW	1	None
Not determined	114	None, *relE-Xre* like, *GNAT-RHH* like, *higBA*, *mazF-RHH* like, *MNT-HEPN* like, *vagCD*, *relBE*, *Bro-Abr* like, *Bro-ArsR* like, *COG5654-COG5642*, *pfam13420-TIGR01764*, *higBA*, *mosTA*, *pfam00583-pfam00376*

* The TAS listed are all the TAS found on plasmids of that Inc type. Individual plasmids may have none, one or a combination of the listed TAS. For a more detailed distribution of the TAS on each individual plasmid, please see S1 Table.

** None = no type II TAS found on the plasmid.

**Fig 1:**
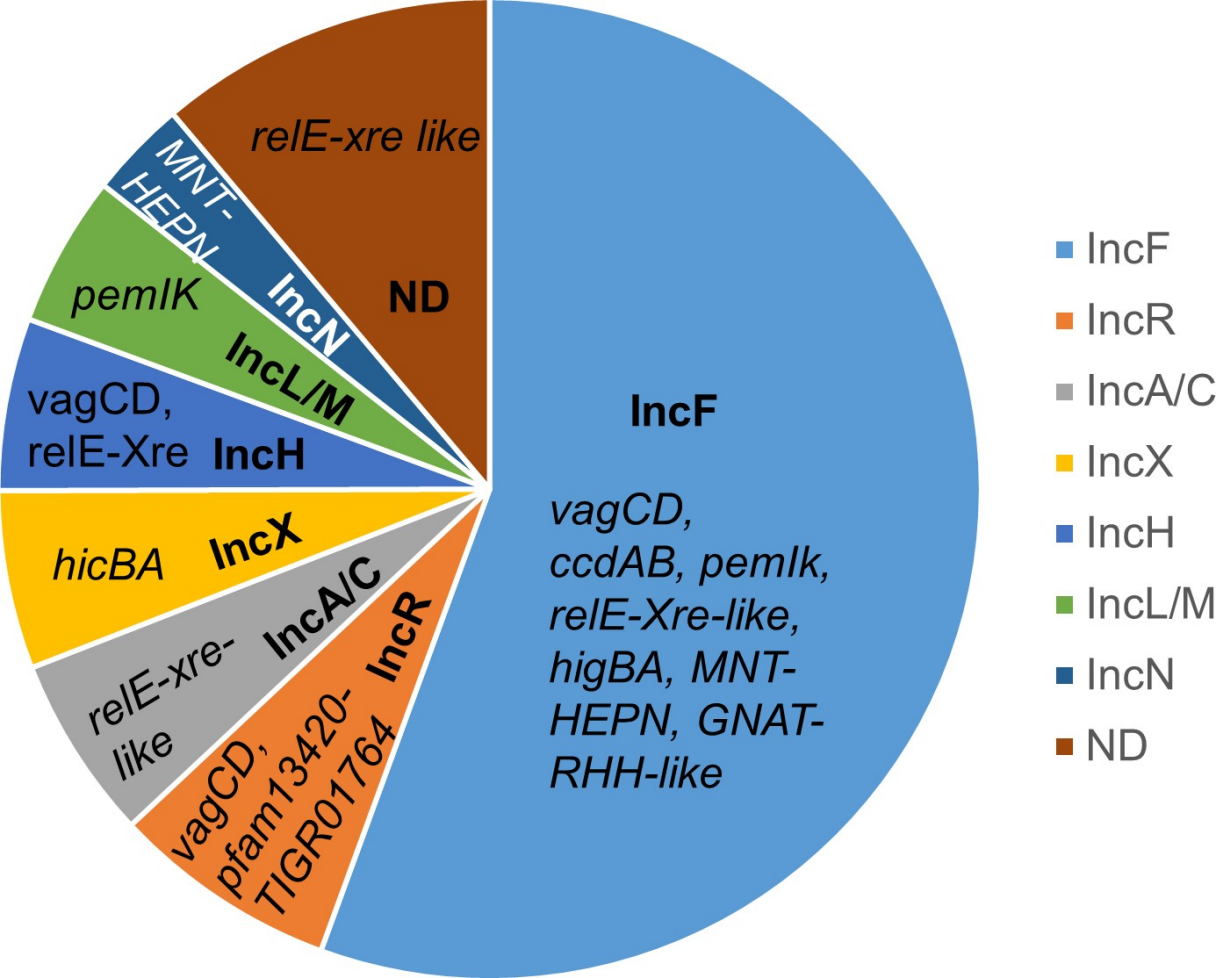
Commonly associated TAS in the major plasmid types (Inc) in *K*. *pneumoniae*. Plasmid incompatibility types are referred to as Inc and ND refers to plasmids that could not be typed by Plasmid Finder. The TAS commonly found in each plasmid type are shown in italics.

Plasmids found in *K*. *pneumoniae* species belonged to 11 different Inc types (Table 4), although around 10% (114/1063) were not assigned an Inc type in PlasmidFinder. More than half of the plasmids were from the IncF replicon group (Fig 1), which is the most common plasmid replicon type in *Enterobacteriaceae* [49]. Replication gene variation can be used to further subdivide IncF plasmids (e.g. IncFIA, IncFIB, IncFIC, IncFII etc) and IncFII plasmids are usually further subdivided with a subscript to indicate the typical host species, e.g. IncFII_K_ and IncFII_S_ for the IncFII plasmids found in *Klebsiella* and *Salmonella* species, respectively. For simplicity, all IncF plasmid subtypes found in *K*. *pneumoniae* strains were grouped as IncF in this study. A number of *K*. *pneumoniae* plasmid types (L, M, X, A, C, N and H) (Table 4, Fig 1) are also commonly found in *E*. *coli* and other *Enterobacteriaceae*, [50] but some that are quite commonly reported in *K*. *pneumoniae* (e.g. IncR, representing 76/1083 plasmids) are rarely reported in *E*. *coli*.

Other associations are noteworthy. For example, *ccdAB* is somewhat specific to IncF plasmids in *K*. *pneumoniae*, while *pemIK* is common to both IncL/M and IncF plasmids (Table 4, Fig 1, S1 Table), in line with previous observations [19]. *vagCD* is another TAS predominantly found on the *bla*_CTX-M_-carrying IncF plasmids in *E*. *coli* (also as previously noted) [21], and in *K*. *pneumoniae* (Table 4, Fig 1). However, *vagCD* was also common in IncH (37/58) and IncR (35 of 76) plasmids in *K*. *pneumoniae* but not in *E*. *coli*. Most IncX plasmids lacked an easily identifiable TAS, save for a few carrying *hicBA*, also as previously noted [51].

### *ccdAB* sequences differ between species, while *pemIK* is highly conserved

*ccdAB* and *pemIK* were the two most predominant TAS modules across both *E*. *coli* and *K*. *pneumoniae* plasmids (Table 4, [21]), and were thus chosen for further study.

**Fig 2:**
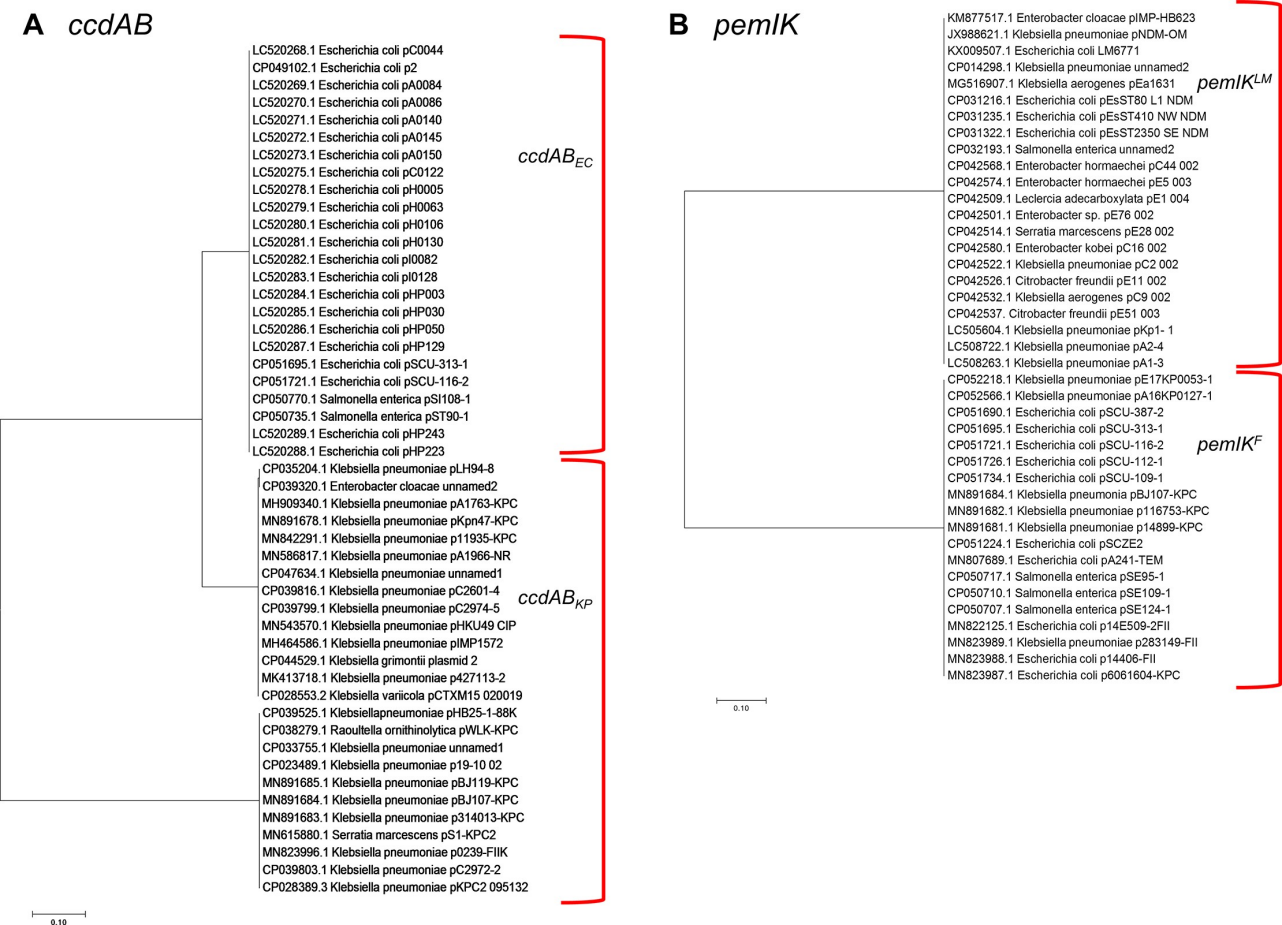
Phylogenetic trees of (A) *ccdAB* and (B) *pemIK*. Representative sequences were used as queries in BLAST searches. Randomly chosen examples of the top 50 resulting hits were then aligned in MEGA7 using the ClustalW algorithm and phylogenetic trees constructed using the Maximum Likelihood Method.

Representative sequences of *ccdAB* were chosen from IncF plasmids derived from two species (*E*. *coli* and *K*. *pneumoniae*; henceforth termed “*ccdAB*_*EC*_*” and “ccdAB*_*KP*_*”* respectively), while variants of *pemIK* were chosen from two plasmid incompatibility types, IncL/M and IncF (henceforth termed “*pemIK*^*LM*^*”* and “*pemIK*^*F*^*”* respectively). These sequences were confirmed as representative of their specific variant by phylogenetic analysis (Fig 2). *ccdAB* shows two clear groups, with one dominated by *E*. *coli* and one by *K*. *pneumoniae*, representing *ccdAB*_*EC*_ and *ccdAB*_*KP*_ respectively (Fig 2A). Each variant was also found, in rare instances, in other species, however given that these are self-transmissible conjugative plasmids, this is to be expected. Similarly, *pemIK* also branched into two groups, with one being dominated by IncF plasmids, and the other by IncL/M plasmids (Fig 2B), irrespective to species. These results confirm that each variant chosen is representative of their specific group.

**Fig 3:**
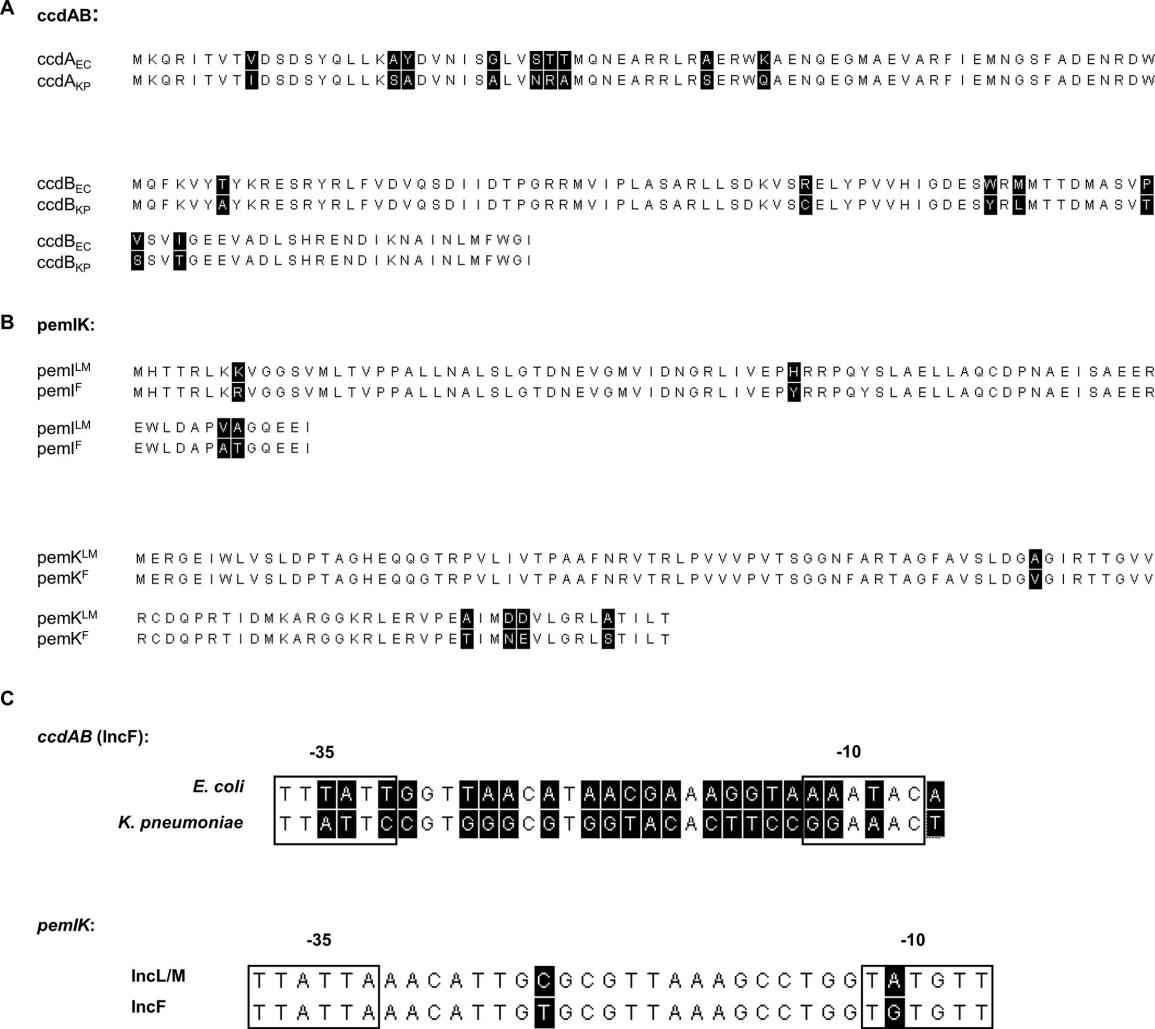
Sequence alignments of *ccdAB* and *pemIK*. These include the amino acid sequence alignments of (**A**) the antitoxin CcdA and the toxin CcdB, from *E*. *coli* and *K*. *pneumoniae*; and (**B**) the antitoxin PemI and the toxin PemK, from IncL/M and IncF plasmids; and (**C**) the nucleotide alignments of the putative promoter regions of the selected TAS. The putative -35 and -10 elements are boxed. All sequences were aligned in MEGA7 using the ClustalW algorithm. Non-identical residues/bases are highlighted in black.

Alignment of *ccdAB*_*EC*_ and *ccdAB*_*KP*_ revealed 85% identity in the nucleotide coding sequence (S1A Fig), translating to 75% and 93% amino acid sequence identities in the antitoxin and toxin respectively (Fig 3A). Predicted secondary structure analysis indicated that although the antitoxin CcdA had the same basic structure in both cases, the toxin CcdB derived from *K*. *pneumoniae* had an additional small α-helix near the C-terminal end (S2 Fig). The putative promoters were less similar, with only a 33% identity throughout the promoter region (-10, -35 and spacers) (Fig 3C). Thus, it is expected that the expression of these TA modules in different species would vary. It has previously been reported that differences in the promoter regions of chromosomal and plasmid mediated *ccdAB* variants was associated with differences in expression and function [32].

By contrast, *pemIK* sequences differed with the plasmid Inc types, rather than with the host bacterial species. Alignment of *pemIK*^*LM*^ *pemIK*^*F*^ revealed 92% identity in the nucleotide coding sequence (S1B Fig), translating to 95% amino acid sequence identities in both proteins (Fig 3B). The PemI antitoxins and PemK toxins also had identical secondary structures (S3 Fig). Similarly, the putative promoters had 94% nucleotide identity (Fig 3C) with only one SNP in the -10 sequences, indicating that *pemIK* TAS is quite conserved within different plasmid types.

### Variation in expression of *ccdAB* in different host strains is greater than expression of *pemIK*

**Fig 4:**
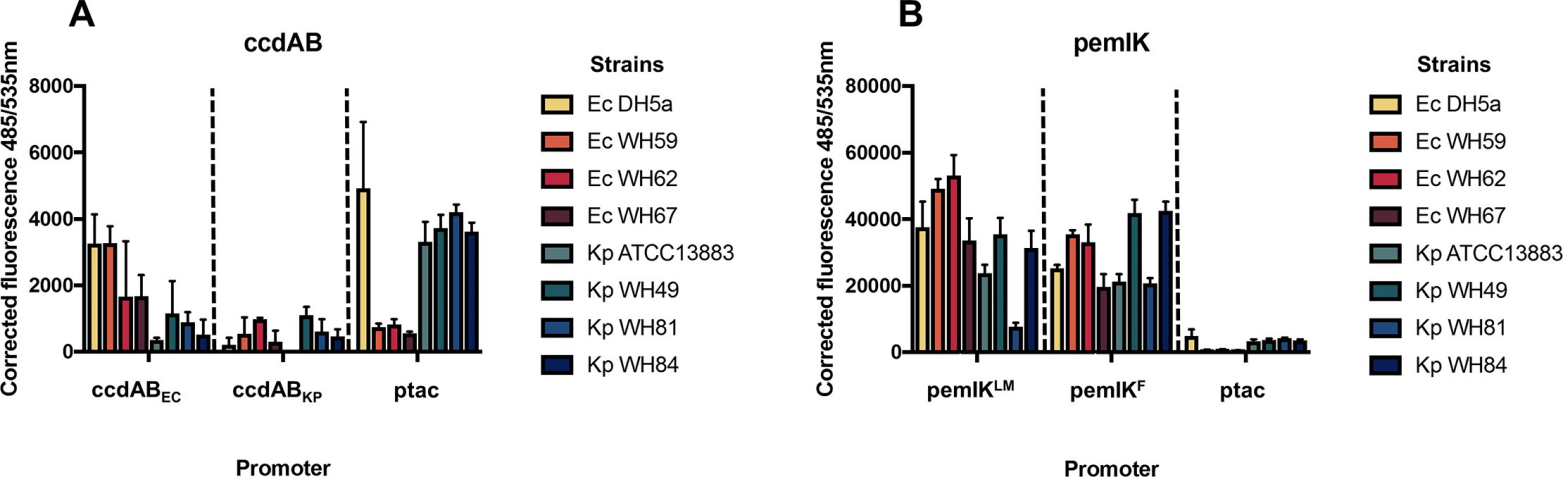
Relative expression of GFP from promoters of (A) *ccdAB* and (B) *pemIK*. The putative promoter of each TAS was inserted upstream of promoterless *gfp* in pANT3 and transformed into four *E*. *coli* and four *K*. *pneumoniae* strains. pANT5, with a constitutive *tac* promoter (ptac)-*gfp* construct, served as a positive control, and values are corrected for background noise. Data shown are the means of three replicates, with the error bars representing one standard deviation from the mean. Please note the ten-fold differences in y-axis scales.

GFP expression from both *pemIK* promoter variants were relatively strong in all strains/species tested, with expression higher than from either of the *ccdAB* promoters tested, as well as the workhorse *tac* promoter [52] (Fig 4), in line with the apparently minor variation in *pemIK* promoters. *pemIK* is the sole TAS evident in published IncL/M plasmids [19], and these data suggest that *pemIK* alone is sufficient to provide stability to the plasmids carrying them, regardless of the host bacterial species.

In contrast, expression of GFP from *ccdAB* promoters is generally lower than that of *pemIK* and showed host species specificity. GFP expression from the *E*. *coli* specific *ccdAB* promoter was higher in *E*. *coli* than in *K*. *pneumoniae* strains. The expression of GFP from the *K*. *pneumoniae* specific *ccdAB* promoter also varied between strains in each species but was relatively lower than from all other promoters tested here. The association between differences in expression of *ccdAB* from different genetic contexts with differences in function in *E*. *coli* has previously been noted [32].

### *ccdAB* function is specific to host strain, whereas *pemIK* is not

**Fig 5:**
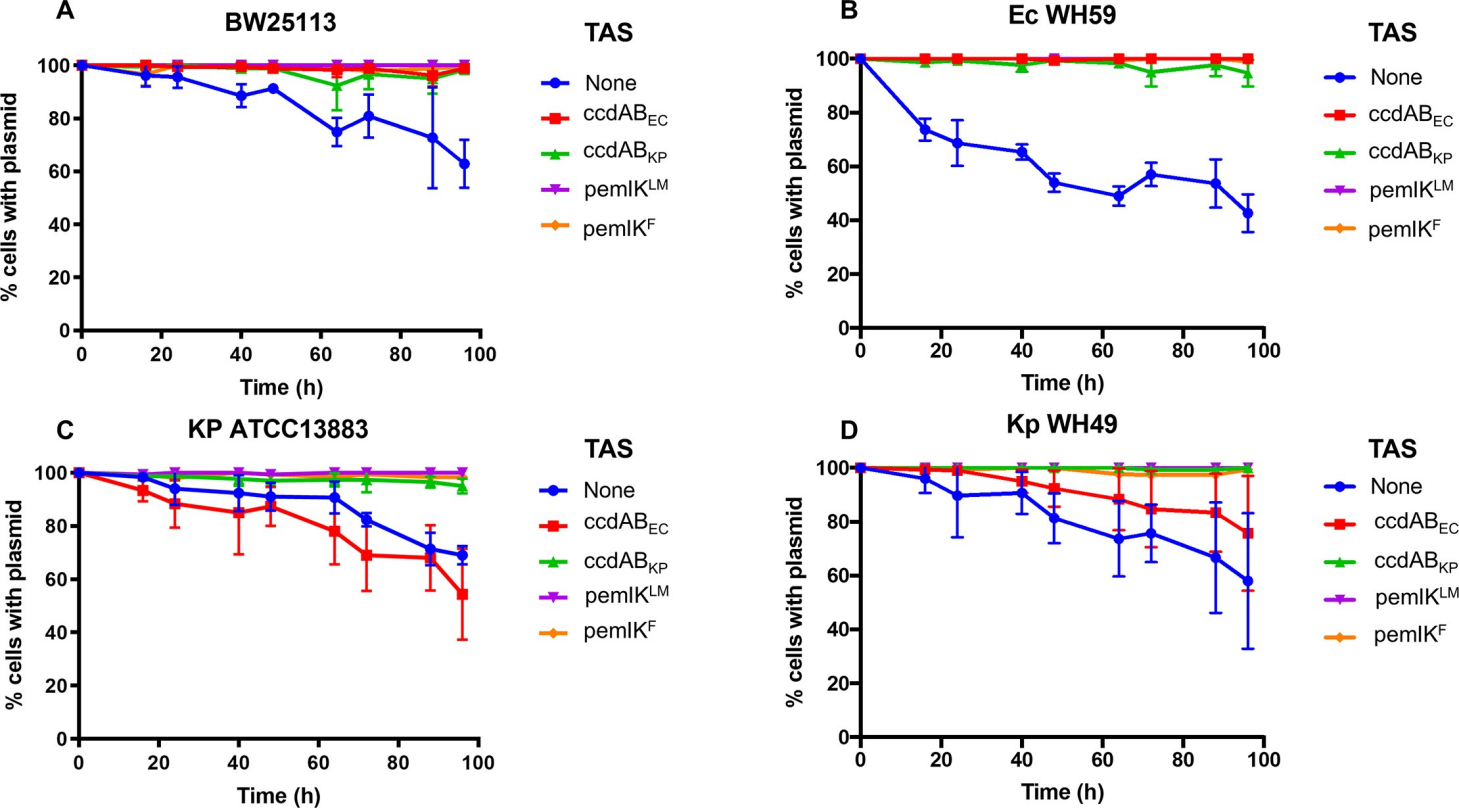
Stability of a low copy number plasmid with and without the specified TAS over 96 h. Each TAS was cloned into a pACYC184 backbone, and the percentage of cells retaining the plasmid calculated at each time point. Measurements were done in two species, *E*. *coli* (**A**: Ec BW25113 and **B**: Ec WH59) and *K*. *pneumoniae* (**C**: Kp ATCC13883 and **D**: Kp WH49). Data shown are the means of three replicates, with the error bars indicating one standard deviation from the mean.

To assess whether the apparent specialisations within *ccdAB* (by host species) and *pemIK* (by plasmid type) are also reflected functionally, we assessed plasmid stability in two host strains of each species, in both low and high copy number plasmids. The low copy plasmid data are presented (Fig 5) as the natural plasmids carrying these TAS are generally low copy number. Consistent data for high copy plasmids can be found in the supplementary material (S4 Fig).

The plasmid stability conferred by *ccdAB* variants varies between host species (Fig 5) and is generally consistent with the GFP expression data, with *ccdAB*_*EC*_ stabilising plasmids in both *E*. *coli* strains but not in *K*. *pneumoniae* strains. *ccdAB*_*KP*_, on the other hand, appears to be less specialised, conferring some plasmid stability in all strains, regardless of species. Given the relatively low level of expression from *ccdAB* promoters and discrepancies in the conferred plasmid stability across species and strain, it is perhaps unsurprising that it is typically one of several TAS in IncF plasmids [21]. By contrast, the more reliably and vigorously expressed *pemIK* variants conferred significant plasmid stability in all strains tested (Fig 5).

Overall, *pemIK* appears less specialised than *ccdAB*. The sequences and expression of *pemIK* variants differed relatively less, and plasmid stability functions of both variants were more consistent. In contrast, *ccdAB* appears to be more variable in sequence and function, different variants conferring plasmid stability in different strains.

*ccdAB* is one of the most well studied TAS and has been reported on both plasmids and chromosomes of different bacterial species [33, 34]. Their sequences and functions also varied based on their genetic context or source of origin (plasmids and bacterial species), and functional differences between plasmid-mediated and chromosomal *ccdAB* have been demonstrated previously [32]. The finding that the CcdB toxin found in the *Salmonella* virulence plasmid pSLT, which has a single amino acid substitution (R99W), is non-toxic and unable to provide plasmid maintenance [53], suggests that even minor variations in TAS protein sequences may lead to changes in their functions. It has also been demonstrated that the antitoxin CcdA originating from one source does not necessarily protect bacteria from toxicity mediated by the toxin CcdB from a different origin, even though both toxins followed the same mechanism of action. For example, CcdA from *Vibrio fischeri* superintegron could not protect from the toxicity of plasmid mediated CcdB_F_ [54].

The inability of *ccdAB* to confer significant plasmid stability in certain host strains raises the possibility that it is performing another role in these strains. It would be of interest to investigate whether these plasmid-mediated *ccdAB* variants are involved in other known functions of TAS within these strains, such as persister formation, antibiotic and heat tolerance, and other bacterial stress responses [10, 12, 15, 33, 55, 56]. Within *E*. *coli*, both an IncF plasmid mediated *ccdAB* and chromosomally located variant have previously been shown to be able to confer some level of protection during antibiotic treatment [33], and the toxin CcdB from IncF plasmid has also been linked to increases in persister cell formation [16].

Variation in *ccdAB* has previously been noted to influence the spread of certain *Salmonella* serovars [53], and we believe that the variation in expression and plasmid stability function of *ccdAB* and *pemIK* may contribute to and influence the epidemiology of the conjugative antibiotic resistance plasmids which carry them. It is hoped that an increased understanding of these effects may be able to aid in the surveillance of these plasmids and the spread of the resistance genes carried by them.

## Supporting information

S1 Table Distribution of type II TA systems in the plasmids of *K*. *pneumonia*. (XLSX)

S1 Fig The alignment and comparison of the nucleotide sequences of *ccdAB* and *pemIK*. Nucleotide sequence alignments of the coding region of (**A**) *ccdAB* from plasmids found in *E*. *coli* and *K*. *pneumoniae*, and (**B**) *pemIK* from IncL/M and IncF plasmids. Sequences were aligned in MEGA7 using the ClustalW algorithm. Non-identical residues are highlighted in black.
(TIF)

S2 Fig Comparison of the predicted secondary structures of *ccdAB*. The predicted secondary structure of the *ccdAB* encoded toxins and antitoxins from plasmids found in *E*. *coli* and *K*. *pneumoniae*. Arrows represent β-strands, cylinder shapes represent α-helices and lines represent random coils. Pred: predicted secondary structure; AA: amino acids; numbers below each structure represent the amino acid positions within the proteins.
(TIF)

S3 Fig Comparison of the predicted secondary structures of *pemIK*. The predicted secondary structure of the *pemIK* encoded toxins and antitoxins from IncL/M and IncF type plasmids. Arrows represent β-strands, cylinder shapes represent α-helices and lines represent random coils. Pred: predicted secondary structure; AA: amino acids; numbers below each structure represent the amino acid positions within the proteins.
(TIF)

S4 Fig Plasmid stability effects of *ccdAB* and *pemIK* in a high copy plasmid. Stability of a high copy number plasmid with and without TAS over 72 h. Each TAS was cloned into a pBCSK+ backbone, and the percentage of cells retaining the plasmid calculated at each time point. Measurements were done in two species, *E*. *coli* (**A**: Ec BW25113 and **B**: Ec WH59) and *K*. *pneumoniae* (**C**: Kp ATCC13883 and **D**: Kp WH49). Data shown are the means of three replicates, with the error bars indicating one standard deviation from the mean.
(TIF)
