# Supplementary figures and images for: Specialised functions of two common plasmid mediated toxin-antitoxin systems, ccdAB and pemIK, in Enterobacteriaceae
Source: PLoS One. 2020 Jun 30;15(6):e0230652. doi: 10.1371/journal.pone.0230652 (PMC7326226; doi:10.1371/journal.pone.0230652)

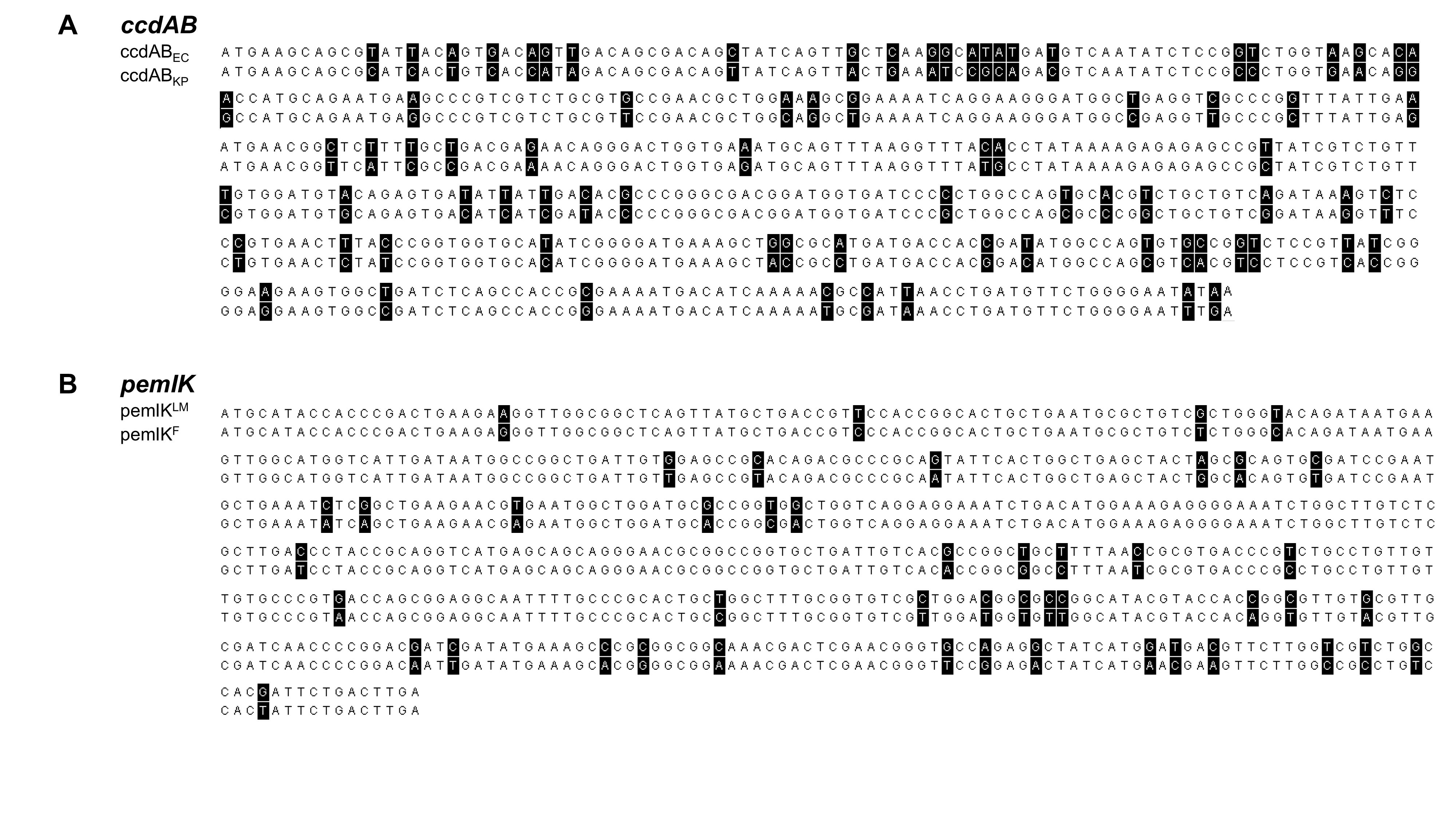

Supplement: S1 Fig — Nucleotide sequence alignments of the coding region of (A) ccdAB from plasmids found in E. coli and K. pneumoniae, and (B) pemIK from IncL/M and IncF plasmids. Sequences were aligned in MEGA7 using the ClustalW algorithm. Non-identical residues are highlighted in black. (TIF) [file pone.0230652.s002.tif]

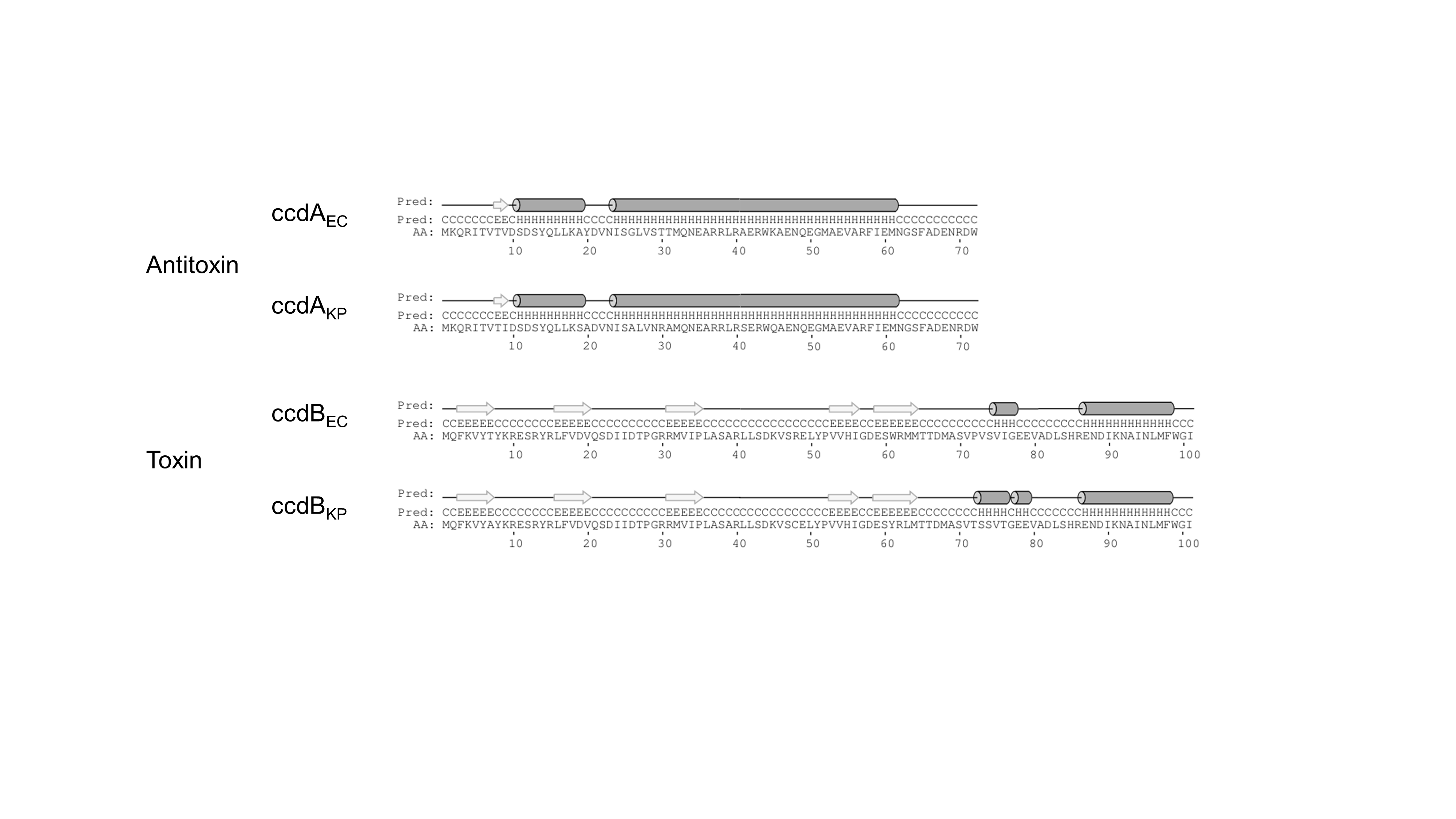

Supplement: S2 Fig — The predicted secondary structure of the ccdAB encoded toxins and antitoxins from plasmids found in E. coli and K. pneumoniae. Arrows represent β-strands, cylinder shapes represent α-helices and lines represent random coils. Pred: predicted secondary structure; AA: amino acids; numbers below each structure represent the amino acid positions within the proteins. (TIF) [file pone.0230652.s003.tif]

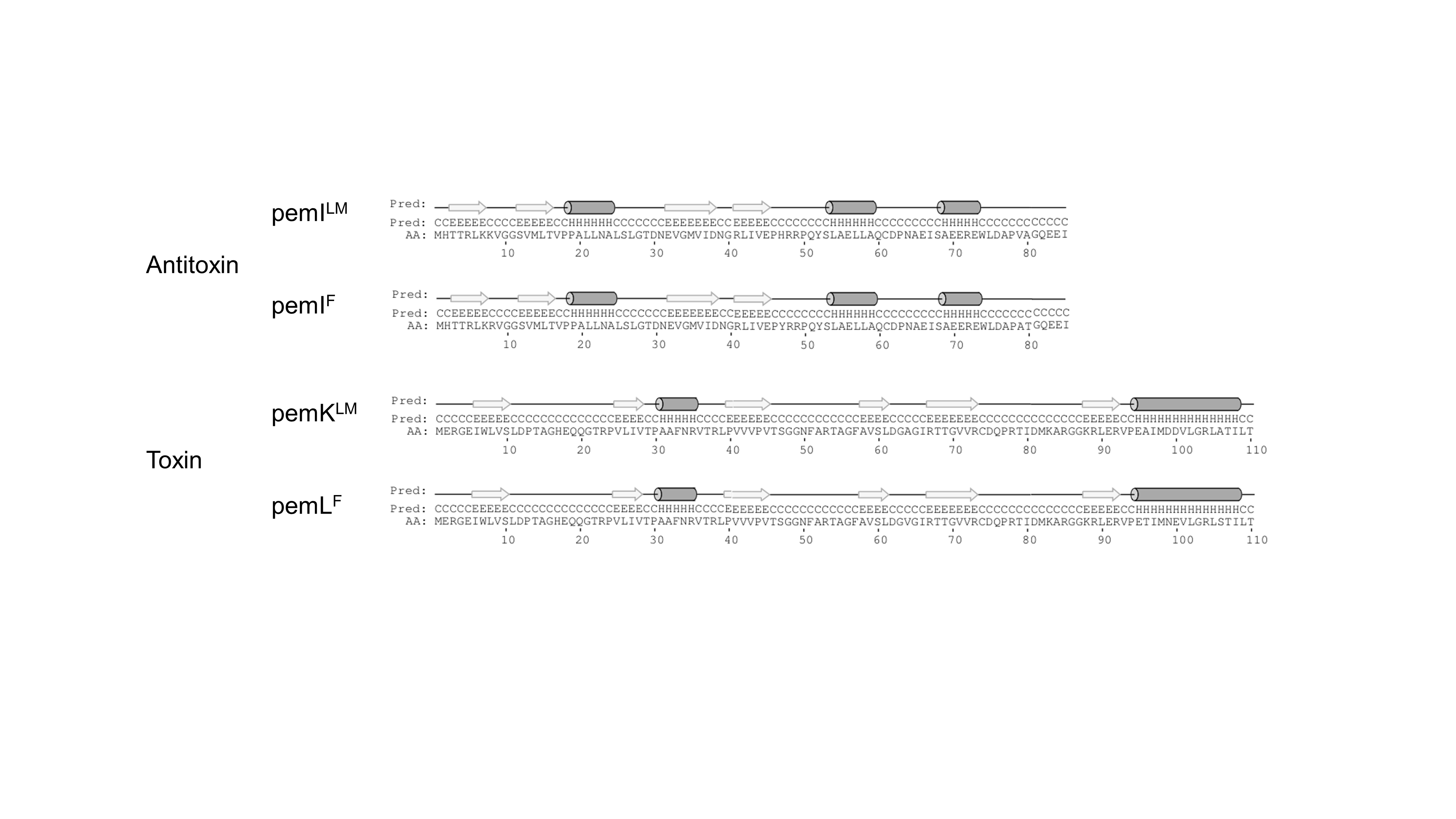

Supplement: S3 Fig — The predicted secondary structure of the pemIK encoded toxins and antitoxins from IncL/M and IncF type plasmids. Arrows represent β-strands, cylinder shapes represent α-helices and lines represent random coils. Pred: predicted secondary structure; AA: amino acids; numbers below each structure represent the amino acid positions within the proteins. (TIF) [file pone.0230652.s004.tif]

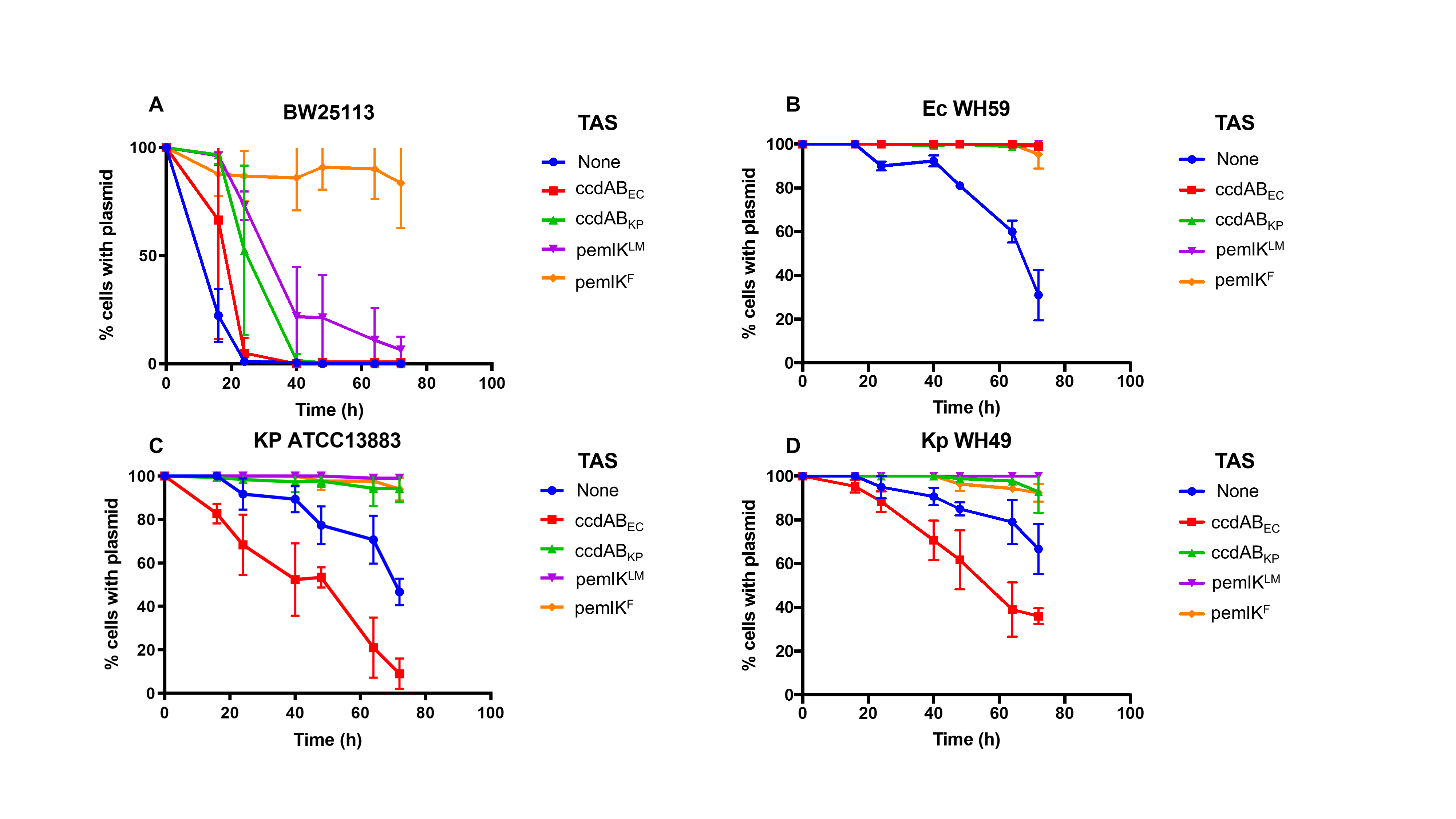

Supplement: S4 Fig — Stability of a high copy number plasmid with and without TAS over 72 h. Each TAS was cloned into a pBCSK+ backbone, and the percentage of cells retaining the plasmid calculated at each time point. Measurements were done in two species, E. coli (A: Ec BW25113 and B: Ec WH59) and K. pneumoniae (C: Kp ATCC13883 and D: Kp WH49). Data shown are the means of three replicates, with the error bars indicating one standard deviation from the mean. (TIF) [file pone.0230652.s005.tif]
